# Screw migration and oesophageal perforation after surgery for osteosarcoma of the cervical spine

**DOI:** 10.1186/s12891-017-1906-5

**Published:** 2017-12-29

**Authors:** Luca Denaro, Umile Giuseppe Longo, Alberto Corrado Di Martino, Nicola Maffulli, Vincenzo Denaro

**Affiliations:** 1 Neurosurgery, Department of Neurosciences DNS, University Hospital of Padova, Padova, Italy; 2 Department of Orthopaedic and Trauma Surgery, Campus Bio-Medico University, Via Alvaro del Portillo, 200, 00128 Trigoria, Rome, Italy; 3 Department of Musculoskeletal Disorders, University of Salerno School of Medicine and Surgery, Salerno, Italy; 4 Centre for Sport and Exercise Medicine, Queen Mary University of London, Mile End Hospital, London, UK

**Keywords:** Osteosarcoma, Cervical spine, Pitfalls, Oesophageal perforation

## Abstract

**Background:**

Even though internal fixation has expanded the indications for cervical spine surgery, it carries the risks of fracture or migration, with associated potential life threatening complications. Removal of metal work from the cervical spine is required in case of failure of internal fixation, but it can become challenging, especially when a great amount of scar tissue is present because of previous surgery and radiotherapy.

**Case presentation:**

We report a 16 year old competitive basketball athlete who underwent a combined anterior and posterior approach for resection of an osteosarcoma of the sixth cervical vertebra. Fourteen years after the index procedure, the patient eliminated spontaneously one screw through the intestinal tract via an oesophageal perforation and developed a severe dysphagia. Three revision surgeries were performed to remove the anterior plate because of the great amount of post-surgery and post-irradiation fibrosis.

**Conclusions:**

Screw migration and oesophageal perforation after cervical spine surgery are uncommon potentially life-threatening occurrences. Revision surgery may be challenging and it requires special skills.

## Background

Only 0.85–4.0% of all osteosarcomas are located in the spine [1–3], and primary osteosarcoma of the cervical spine is even rarer [4, 5], with less than 50 patients reported in the literature [6]. In the last two decades, aggressive adjuvant and neoadjuvant therapy have improved the outcome of osteosarcoma patients [7, 8]. Generally, the prognosis for patients with spinal osteosarcoma is worse compared with the prognosis of patients with osteosarcoma of the extremities [2, 9–13].

Internal fixation has expanded the indications for cervical surgery [14], and allows to reliably achieve fusion in cervical spine surgery and reduce graft-related complications [15]. However, metal work carries the risks of fracture or migration, with associated compression of the spinal cord, paralysis or neural injury, oesophageal penetration, tracheal impingement with airway obstruction, mediastinitis, and death [16–22]. Failure of internal fixation requires removal of metal work from the cervical spine, which may become challenging, especially when a great amount of scar tissue is present because of previous surgery and radiotherapy.

We report a 16 year old competitive basketball athlete who underwent a combined anterior and posterior approach for resection of an osteosarcoma of the sixth cervical vertebra. Fourteen years after the index procedure, the patient eliminated spontaneously one screw through the intestinal tract via an oesophageal perforation and developed a severe dysphagia. Three operations were necessary to remove the anterior plate.

Our patient was informed that data concerning his case would be submitted for publication, and gave written consent for this.

## Case presentation

**Fig. 1:**
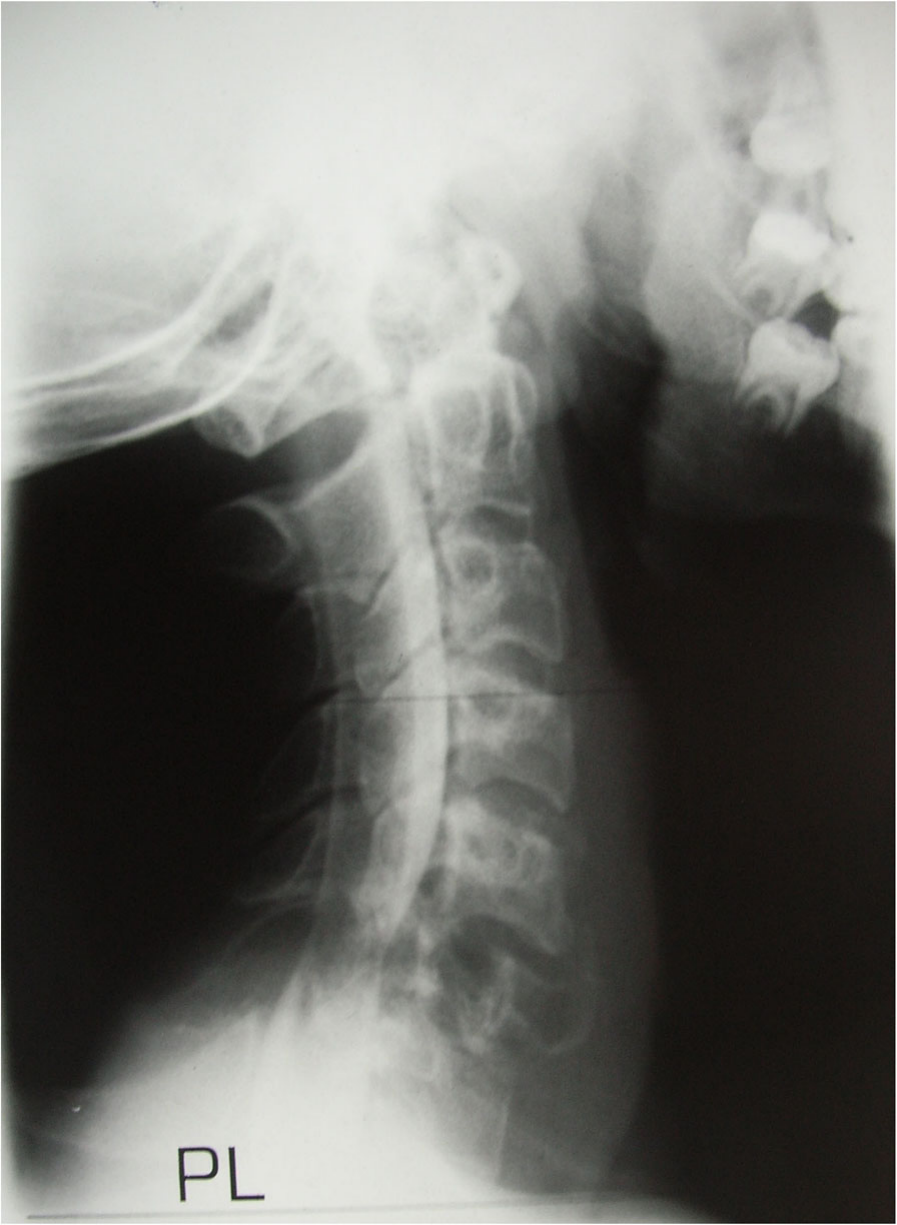
Lateral radiograph of the cervical spine of the young athlete showing the osteolytic area of C6

In October 1983, a 16 year old male basketball player presented to another hospital with a 1 month history of paresthesiae of the index and long finger of the right hand. The patient was diagnosed with tendinopathy of the wrist extensor tendons, and managed for 2 months with physiotherapy, anti-inflammatory drugs and rest. In January 1984, the young athlete presented to the emergency room of our hospital with persistent neck pain after twisting his neck during a basketball game. His initial examination showed contracture of the cervical paravertebral muscles. Neurological examination was unrewarding. Radiographs showed an osteolytic area of the sixth vertebra of the cervical spine (C6) (Fig. 1). Myelograms showed compression of the cord. A CT scan showed a mass involving the right side of the vertebra including the body, the lamina, and the lateral mass with its joint. The patient was immobilized in a halo cast, and referred to the senior author (V.D.), who performed a surgical resection of the mass through a staged anterior and posterior approach.

The first stage was performed with a posterior approach with the patient prone. At surgery, the tumour was removed posteriorly. The tumour involved the right side of the vertebra including the body, the lamina, and the lateral mass of C6. An intra-operative biopsy was performed, and a histopathological diagnosis of osteosarcoma was formulated.

A wide resection of the mass was performed. A Roy Camille plate was used on the left side, which was free of tumour. A non-instrumented fusion was obtained on the right side with a bony bar harvested from the ipsilateral iliac crest. The bar was secured by screws onto the lateral articular masses of C4 and T1.

**Fig. 2:**
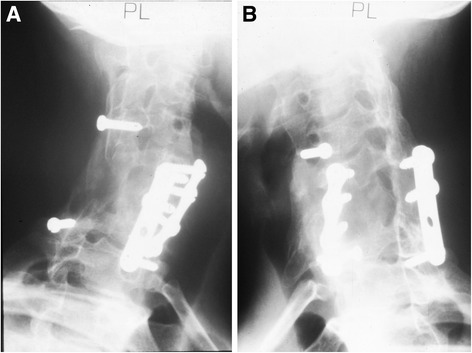
**a-b** Oblique radiographs of the cervical spine showing a Roy Camille plate positioned to obtain posterior fusion on the left intact side, and 2 screws on the lateral articular masses of C4 and T1 to maintain the graft. An anterior plate stabilized the anterior aspect of the cervical spine

Two weeks after the first procedure, the patient underwent a further operation through an anterior approach for complete removal of the neoplasm and fusion. A complete excision of the anterior portion of the vertebral body was performed. After wide resection, the body was reconstructed anteriorly with an iliac crest autograft and a plate with screws (Fig. 2a-b).

The patient was immobilized in a Halo cast for 4 months, followed by a SOMI (Sternal Occipital Mandibular Immobilizer) brace for 1 year. After surgery, the patient underwent radiotherapy (40 applications). Recovery was uneventful, and the patient returned to his activities of daily living, and qualified as a surveyor. He returned to play competitive basketball in 1989 for 2 seasons.

In 1993, the patient started to complain of mild dysphagia, but all the physicians attributed this to post-surgery and post-irradiation adherences.

**Fig. 3:**
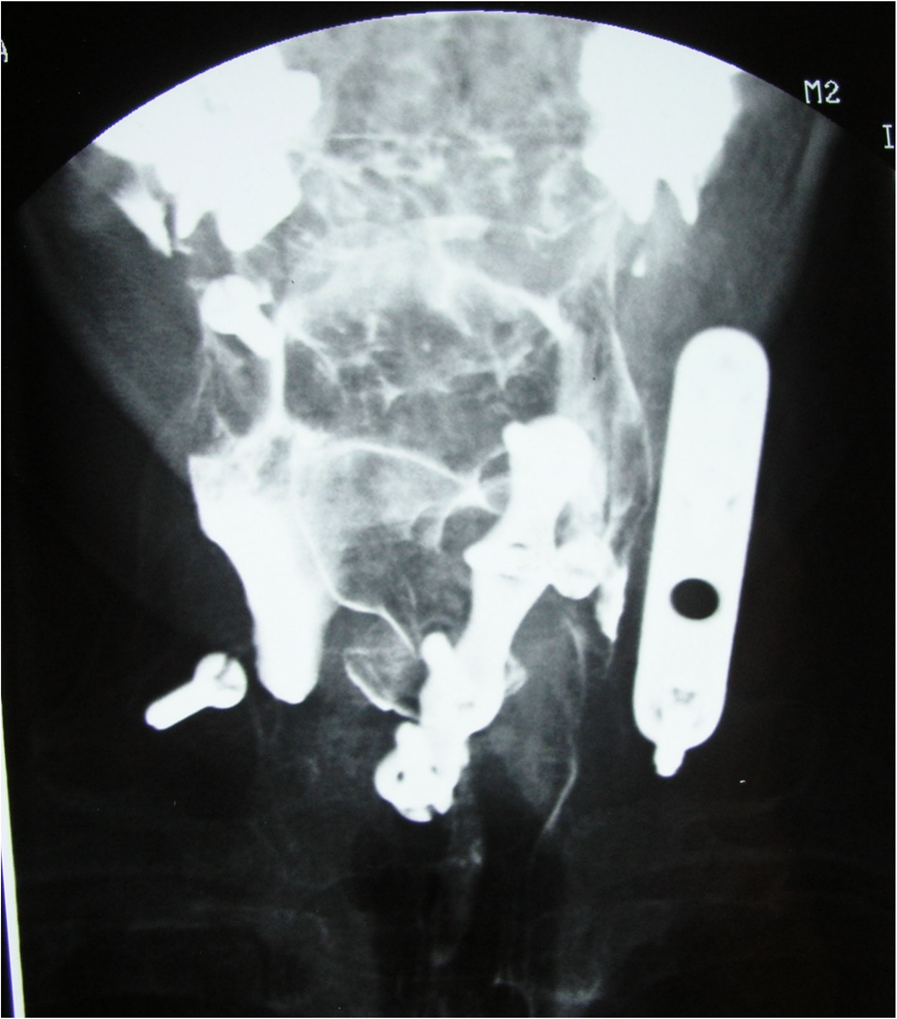
Fluoroscopy showing the second screw of the anterior plate being partially extruded

**Fig. 4:**
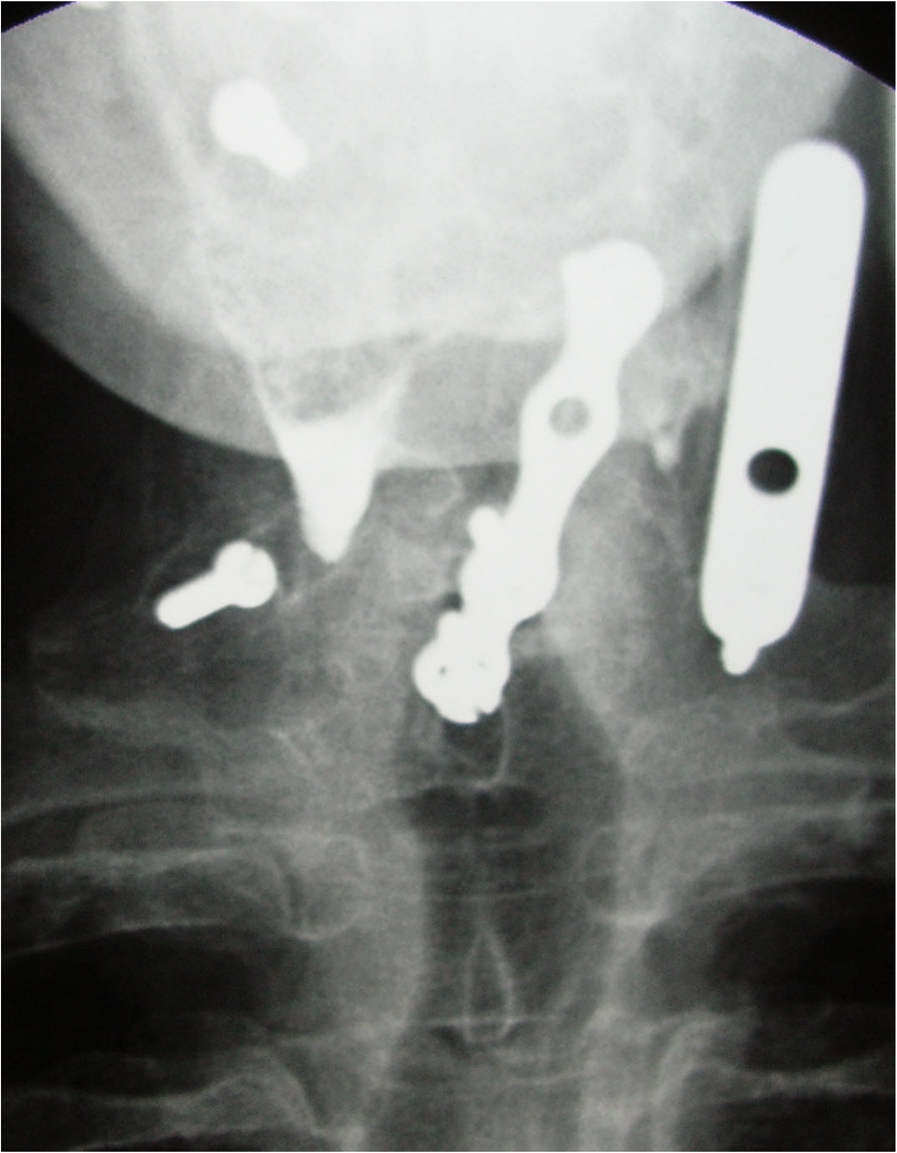
Radiograph showing the absence of the mobilized screw in the anterior plate

**Fig. 5:**
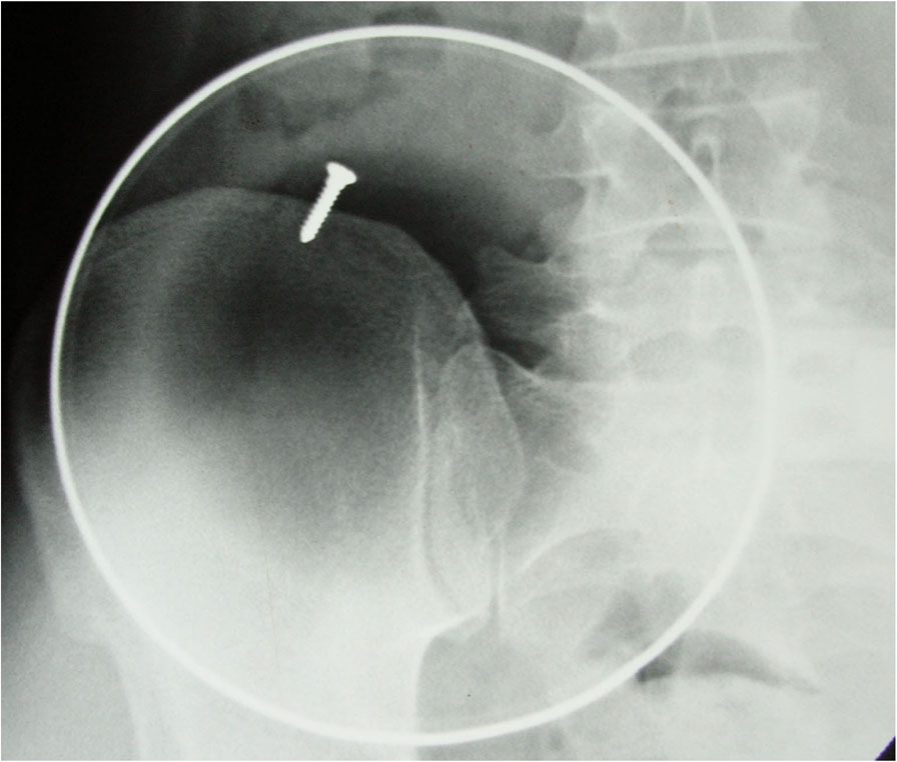
Radiographs showing the screw migrated in the colon

In July 1998, the patient underwent fluoroscopy for the diagnosis of the dysphagia, showing the second screw of the anterior plate being partially extruded (Fig. 3). One month later, in August 1998, the patient complained of complete inability to swallow. Radiographs showed the absence of the mobilized screw in the anterior plate (Fig. 4), and the screw migrated in the colon (Fig. 5). The patient underwent a colonoscopy, but the endoscopist was not able to remove the screw. Two days after the colonoscopy, the patient eliminated spontaneously the screw through the intestinal tract.

The patient underwent a first operation for removal of metal work through the same right pre-sternocleidomatoid approach in another hospital. During the operation, removal of the plate was not possible for the presence of much post-surgery and post-irradiation fibrosis.

**Fig. 6:**
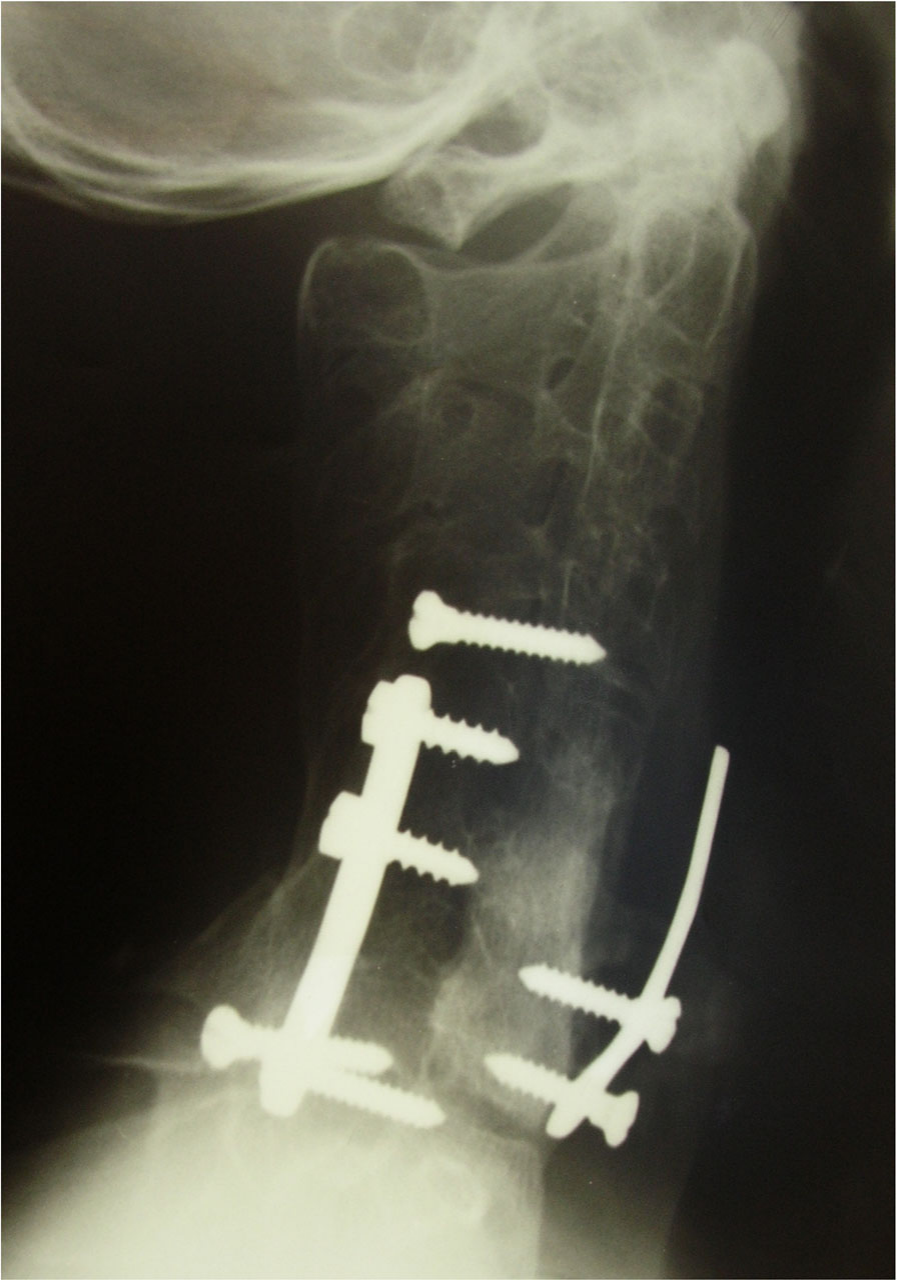
During the second operation for surgical removal of metal work through a wide U approach, it was possible to remove only 1 screw

In February 2001, the patient underwent a second operation for surgical removal of metal work through a wide U approach with an ear, nose, and throat surgeon in the same hospital, but it was only possible to remove 1 screw (Fig. 6). During surgery, a tracheostomy was performed, and closed after 2 weeks.

The patient continued to complain of dysphagia for 1 year, and he lost 15 Kg. In January 2001, the patient returned to the senior author, weighing 45 Kg. The patient underwent total parenteral nutrition for 5 months, and returned to his normal weight of 60 Kg.

On July 1st 2001, the patient underwent a third operation, this time performed by the senior author, with another ear, nose, and throat surgeon to tackle any intra-operative oesophageal injury. During the operation, no signs of oesophageal perforation became evident. At surgery, much fibrosis was found. To reach the vertebral plane, MESNA (sodium 2-mercaptoethanesulfonate) (Uromitexan, Bristol) was intra-operatively applied on the fibrous tissue to ease tissue dissection. A modified dissector was used to release the drug locally [23]. Duration of local application was about 20–30 s; repeated applications were performed during surgery. Average volume of agent used was about 10cm^3^.

Removal of the plate proceeded uneventfully, and a suction drain was left in situ after the procedure. The patient was immobilized in a Philadelphia collar for 2 months. Post-operative recovery was uncomplicated.

**Fig. 7:**
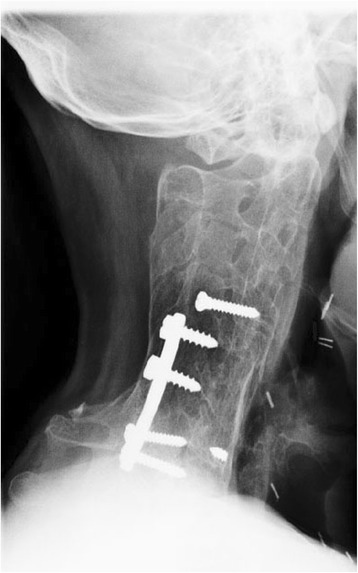
Lateral 24 year follow up radiographs of the cervical spine of the patient

At 24 year from the first operation, the patient is symptom free. When last reviewed in October 2008 (Fig. 7), he had no disability, scoring 0 on the Neck Disability Index [24].

## Discussion

Primary bone tumors are approximately 0,4% of all tumors [25]. Primary spine bone tumors are further rare, accounting approximately 4,2% of primary bone tumors [26]. Moreover cervical spine tumour are less common than tumor that occurs in the torcic and lumbar tract [27]. In the first two decades of live primary spine tumors are often benign; however the incidence of malignant tumors increases with age. Pain is the most common initial symptom. It is worse at night and is not relived with analgesic. Neurological symptoms occur usually late. Radicular symptoms are common, although neurologic findings are infrequent in primary benign tumors. Osteoid osteomas, osteoblastomas, giant cell tumors, eosinophilic granuloma and aneurismal bone cysts are the most common lesions [27]. Treatments depend on the location of the lesion, the presumptive diagnosis and the oncologic stage. Benign tumors such as osteoid osteoma or osteoblastoma can be treated with excisional biopsy or intralesion curettage. However, osteoblastoma and giant cell tumor, given the potential local aggression of the disease and the potential recurrence, can require a marginal excision or radiation therapy when resection is incomplete [28]. Aneurismal bone cyst can require embolization therapy followed by marginal excision. Hemangioma generally requires no treatment; however, when symptomatic, can be treated with embolization therapy. Spine stabilization is required after anterior resection or after multilevel laminectomies but the prognosis for primary benign tumors in good. Malignant tumors are often found in old patients. They often involve the vertebral body and the surrounding tissues, and therefore neurological defects are common. Plasmacitoma, chordoma, Ewing sarcoma, chondrosarcoma and non Hodgking’s lymphoma are frequent spine malignant tumors and often involve several vertebrae [27]. Malignant tumor therapy requires a multidisciplinary approach. Tumor en bloc excision is the most appropriate treatment, often combined with chemotherapy and radiotherapy. Osteosarcoma is a malignant aggressive tumor that often requires surgery, chemotherapy and radiotherapy. The prognosis of primary spine malignant tumors is worse compared with benign tumor, but better compared with bone metastasis. Metastatic lesions of spine are common: they can be found in 30% to 70% of patients with tumors [29]. Malignant tumor therapy requires a multidisciplinary approach. Tumor en bloc excision is the most appropriate treatment, often combined with chemotherapy and radiotherapy. Osteosarcoma is a malignant aggressive tumor that often requires surgery, chemotherapy and radiotherapy. The prognosis of primary spine malignant tumors is worse compared with benign tumor, but better compared with bone metastasis. Metastatic lesions of spine are common: they can be found in 30% to 70% of patients with tumors [26]. The prognosis of patients with metastatic tumors is poor. Considering the low life expectancy, the goal of treatment is the stabilization of cervical spine, pain control and prevention of rapid neurological deterioration. Instability, neurological deterioration or intractable pain in patients with at least 3 mounts of live are often indications for surgery. Primary bone tumors provide reconstructive challenges given that patients have an improved prognosis compared to metastatic lesions and there is a relatively poor fusion rate with these etiologies by radiation therapy [30]. Management of patients with osteosarcoma of the cervical spine is challenging. As no clinical, randomized, prospective trials have been, and are unlikely to be, performed on this condition, there is no consensus about the optimal management. Specifically, the need for and duration of spinal immobilization and the role of adjuvant therapy are frequent clinical dilemmas. Also, a cervical spine osteosarcoma is a diagnostic challenge, especially when it presents with atypical features [6, 31]. The therapeutic goals in these patients are to remove the tumour, achieve spinal stability, preserve neurological function, and relieve pain [32–34].

The wide time span over which the patient was managed inevitably introduces inconsistencies and changes in terms of diagnostic methods, surgical techniques, and aims and expectations of management [35, 36]. However, our follow up of 24 years is very long, and allows to consider that, by then, the results of surgery would have stabilised, and recovery, or worsening and death from the osteosarcoma, would have occurred. Long-term evaluation is necessary, particularly as osteosarcomas can recur.

Internal fixation is regarded as the most common approach to achieve fusion after cervical spine surgery and to reduce graft-related complications [37]. However, metal work carries the risks of fracture or migration, with associated damage to the surrounding structures [16–18].

Oesophageal perforation has been reported as consequence of a graft dislodgment or screw migration [38–40]. Cloward first reported a patient with migration of the graft into the oesophagus, requiring endoscopic removal [41]. Removal of plate and screws was needed for two patients reporting oesophageal problems from screw migration [16]. Migrated screws can be found in the lower gastrointestinal tract of patients who underwent anterior cervical fixation without significant associated morbidity [14, 17, 42, 43], or they can be orally extruded, with patients complaining of swallowing difficulties and dysphagia which resolved immediately after extruding the screw [44, 45].

Our patient had an oesophageal perforation caused by the one displaced cervical screw, with spontaneous elimination through the gastrointestinal tract. Oesophageal perforation is a rare and potentially fatal complication of cervical spine surgery. It can have a benign evolution, and at times is completely asymptomatic [14]. We decided to remove the metal work given the presence of persistent dysphagia and severe weight loss, and not only on the basis of diagnostic imaging, as spontaneous recovery and no oesophageal scarring has been previously identified in similar patients [14]. However, following asymptomatic elimination through the gastrointestinal tract of one screw, and surgical removal of another screw, our patient continued to complain of swallowing difficulties and severe dysphagia.

Removal of metal work can be difficult given the presence of when a great amount of adhesion and scarring because of previous surgery and radiotherapy. MESNA is a clear liquid mucolytic agent which can quickly dissolve connections between tissues [46, 47]. A randomized controlled trial analyzed the effectiveness of MESNA in chemical dissection of peridural fibrosis in patients who underwent revision lumbar spine surgery [23]. MESNA proved effective as chemical dissector for epidural fibrosis in revision lumbar spine surgery, significantly reducing operative complications, with a decrease in surgical time and surgical difficulty [23]. MESNA has been also used as a local adjuvant in chemical assisted dissection in surgery for endometrial cysts [48], abdominal myomectomies [46], and in surgical excision of cholesteatoma matrix, in which neural and bony structures are in contact [49]. MESNA intravenously administration is rarely related to side effects (eg, nausea, vomiting, diarrhea, allergic reactions, hypertension) [48]. The use of MESNA in our patient facilitated removal of the metal work without complications, despite the high quantity of scar tissue.

This article describes a potential life threatening complication of cervical spine surgery and a possible therapeutic strategy in patients with previous surgery and radiotherapy. Particularly we report of a patient who returned to play competitive basketball after surgery and in whom the screw migration was misdiagnosed for about 5 years. Esophageal perforation is rare potentially fatal complication of anterior cervical spine surgery. The diagnosis is difficult due to low overall prevalence of this pathology and vague and variable clinical presentation. Patients may present no signs or symptoms at all but also florid sepsis and respiratory distress. Yee et al. reported a case of a patient who not recalls any symptoms related to dysphagia, odynophagia, neck pain, or cough [50]. A similar case was reported by Pompili et al. who described the disappearance of a screw after 6 months, presuming that the screw have entered the gastrointestinal tract and exited the patient [51]. Our patient, instead, developed a severe dysphagia and malnutrition but eliminated spontaneously the screw through the intestinal tract. Most cases of esophageal perforation are discovered at the time of surgery or during the acute or subacute postoperative period. We report an oesophageal perforation developed 14 years after the index procedure. A number of different treatment options have been reported in literature with varied results and without a consensus on the management of these injuries. Antibiotics, nasogastric placement and esophageal diversion without surgery have a very limited role; surgical exploration with irrigation and debridement, primary repair with multiple flap options are often required.

Moreover this article adds evidence on a difficult post-irradiation surgery in which the intra-operatively application of MESNA was fundamental for fibrous tissue dissection.

## Conclusion

Biopsy is important to confirm the diagnosis of osteosarcoma of the cervical spine in atypical presentations. Neck pain should always be considered potentially dangerous in children: accurate examination and investigations are required to avoid delay in diagnosis and management. Oesophageal perforation can occur as complication of cervical spine surgery. In these patients, the decision of metal work removal should be considered in the presence of clinical symptoms, and not just on the basis of imaging. Lastly, cervical spine revision surgery is challenging. MESNA has been used in lumbar spine revision surgery to try to reduce the operative complications and decrease technical difficulties [23]. Further studies are required to support the efficacy and safety of MESNA also in cervical spine revision surgery.

## Abbreviations

CT: Computed tomography
MESNA: Sodium 2-mercaptoethanesulfonate
SOMI: Sternal occipital mandibular immobilizer
